# Probing Context-Dependent Modulations of Ipsilateral Premotor-Motor Connectivity in Relapsing-Remitting Multiple Sclerosis

**DOI:** 10.3389/fneur.2020.00193

**Published:** 2020-05-05

**Authors:** Elisa Ruiu, Raffaele Dubbioso, Kristoffer Hougaard Madsen, Olivia Svolgaard, Estelle Raffin, Kasper Winther Andersen, Anke Ninija Karabanov, Hartwig Roman Siebner

**Affiliations:** ^1^Danish Research Centre for Magnetic Resonance, Centre for Functional and Diagnostic Imaging and Research, Copenhagen University Hospital Hvidovre, Section 714, Hvidovre, Denmark; ^2^Department of Neurology, University Hospital of Sassari, Sassari, Italy; ^3^Department of Neurosciences, Reproductive Sciences and Odontostomatology, University Federico II of Naples, Naples, Italy; ^4^Section for Cognitive Systems, Department of Applied Mathematics and Computer Science, Technical University of Denmark, Lyngby, Denmark; ^5^Brain Mind Institute and Centre of Neuroprosthetics, Swiss Federal Institute of Technology (EPFL), Geneva, Switzerland; ^6^Department of Nutrition, Exercise and Sports, University of Copenhagen, Copenhagen, Denmark; ^7^Department of Neurology, Copenhagen University Hospital Bispebjerg, Copenhagen, Denmark; ^8^Faculty of Medical and Health Sciences, Institute for Clinical Medicine, University of Copenhagen, Copenhagen, Denmark

**Keywords:** multiple sclerosis, dual-site TMS, fatigue, movement preparation, dorsal premotor cortex, primary motor cortex

## Abstract

**Objective:** We employed dual-site TMS to test whether ipsilateral functional premotor-motor connectivity is altered in relapsing-remitting Multiple Sclerosis (RR-MS) and is related to central fatigue.

**Methods:** Twelve patients with RR-MS and 12 healthy controls performed a visually cued Pinch-NoPinch task with their right hand. During the reaction time (RT) period of Pinch and No-Pinch trials, single-site TMS was applied to the left primary motor cortex (M1) or dual-site TMS was applied to the ipsilateral dorsal premotor cortex (PMd) and to M1. We traced context-dependent changes of corticospinal excitability and premotor–motor connectivity by measuring Motor-Evoked Potentials (MEPs) in the right first dorsal interosseus muscle. Central fatigue was evaluated with the Fatigue Scale for Motor and Cognitive Functions (FSMS).

**Results:** In both groups, single-pulse TMS revealed a consistent increase in mean MEP amplitude during the Reaction Time (RT) period relative to a resting condition. Task-related corticospinal facilitation increased toward the end of the RT period in Pinch trials, while it decreased in No-Pinch trials. Again, this modulation of MEP facilitation by trial type was comparable in patients and controls. Dual-site TMS showed no significant effect of a conditioning PMd pulse on ipsilateral corticospinal excitability during the RT period in either group. However, patients showed a trend toward a relative attenuation in functional PMd-M1 connectivity at the end of the RT period in No-Pinch trials, which correlated positively with the severity of motor fatigue (*r* = 0.69; *p* = 0.007).

**Conclusions:** Dynamic regulation of corticospinal excitability and ipsilateral PMd-M1 connectivity is preserved in patients with RR-MS. MS-related fatigue scales positively with an attenuation of premotor-to-motor functional connectivity during cued motor inhibition.

**Significance:** The temporal, context-dependent modulation of ipsilateral premotor-motor connectivity, as revealed by dual-site TMS of ipsilateral PMd and M1, constitutes a promising neurophysiological marker of fatigue in MS.

## Highlights

- Dynamic modulation of ipsilateral premotor-to-motor cortical drive was probed with TMS.
- Patients with relapsing-remitting multiple sclerosis showed normal modulation during a cued Pinch-NoPinch task.
- Attenuation of premotor-to-motor drive in a NoPinch context scaled positively with motor fatigue in patients.
- Context-dependent attenuation of premotor-to-motor drive may contribute to motor fatigue in multiple sclerosis.

## Introduction

Multiple Sclerosis (MS) is the most common autoimmune disorder of the central nervous system (CNS) (1), and its pathology includes both axonal damage and demyelination (2). A majority of MS patients initially exhibit a relapsing-remitting disease course (RR-MS), characterized by attacks with acutely emerging focal neurological deficits that totally or partially recover over the following weeks. Relapses can cause a large variety of classic neurological deficits, affecting motor and sensory function, but also “less quantifiable” symptoms such as excessive motor or cognitive fatigue (3).

Transcranial magnetic stimulation (TMS) allows corticomotor excitability to be quantified by recording motor evoked potentials (MEP) and is widely used to characterize cortico-motor dysfunction in MS (4, 5). Single-pulse TMS studies have demonstrated abnormal corticospinal excitability and connectivity in patients with RR-MS even during relapse-free periods (6). Single-pulse studies also found that basic MEP-based excitability measures scale with individual motor function or disability scores (7, 8). Paired-pulse TMS, which applies a conditioning and test pulse with the same coil, has been used to probe intracortical excitability in RR-MS and has revealed a link between individual disability and measures of intracortical inhibition and intracortical facilitation (8, 9). These correlations between intracortical excitability and disability score at the single-patient level may be obscured when pooling patients (10), highlighting the importance of taking inter-individual variations into account when investigating a heterogeneous disease like multiple sclerosis (11).

TMS can also be used to investigate the neurobiological mechanisms underlying specific motor symptoms like motor fatigue, which represents one of the most common symptoms in MS (12). One study linked motor fatigue to alterations of intracortical excitability in patients with RR-MS while the patients were at rest (13). Other TMS studies used single-pulse TMS to probe corticospinal excitability during the performance of a simple, visually cued reaction time task. These studies revealed a reduction of pre-movement facilitation that correlated with individual fatigue scores (14, 15), pointing to an impaired initialization of movements. Complementing these task-related TMS studies, task-related functional brain imaging studies found excessive recruitment of higher-order premotor areas, such as the dorsal premotor cortex (16–18). The premotor activity may reflect excessive volitional drive in the context of inefficient movement initiation.

Dual-site TMS (dsTMS) has been successfully used to probe the effective connectivity of pathways projecting from cortical or cerebellar brain regions to the precentral primary motor cortex (19–22). These dsTMS paradigms apply a conditioning stimulus (CS) over the remote motor area and give a test stimulus (TS) over the primary motor hand area (M1-HAND) to probe the effect of the conditioning stimulus on corticospinal excitability. Ipsilateral premotor-to-primary motor connectivity can be probed with optimized small TMS coils, which apply the CS over the dorsal premotor cortex (PMd) and the test stimulus (TS) over ipsilateral M1-HAND (23, 24). Groppa et al. introduced a dsTMS paradigm in which the TS is applied over M1-HAND 0.8–2.0 ms before a CS over ipsilateral PMd (23, 24). The premotor CS facilitates corticospinal excitability in ipsilateral M1-HAND via an ultra-fast premotor-to-motor pathway. Functional premotor-to-motor interaction, as probed by this dsTMS paradigm, dynamically changed depending on the motor context (23, 24). When healthy individuals performed a two-choice Go-NoGo task, premotor-motor connectivity showed a trial-dependent divergence during the late RT period: Go trials led to a dynamic increase, while No Go trails resulted in a dynamic decrease (23).

Building on the work by Groppa et al. (23, 24), we used a slightly modified dsTMS paradigm to examine how movement preparation and movement inhibition dynamically modulate corticospinal excitability and ipsilateral PMd-to-M1 connectivity during the RT period in patients with RR-MS and healthy participants. We also explored whether dynamic changes in functional PMd-to-M1 connectivity during movement initiation or inhibition would scale with subjectively experienced fatigue in MS patients. Since we were interested in the control of dexterous movements, we selected a visually cued pinching task rather than a cued two-choice movement task. We hypothesized that multiple sclerosis would reduce the dynamic modulation of premotor-to-motor facilitation during the RT period and that deficient modulation of premotor-to-motor facilitation in the pre-movement phase would scale with the individual experience of motor fatigue in MS patients.

## Materials and Methods

### Subjects

Fourteen healthy controls (HC) (six men, aged 37.5 ± 10.8 years, mean ± SD) and 14 patients with relapsing-remitting MS (RR-MS) (seven men, aged 37.6 ± 8.5 years, mean ± SD) were enrolled in the TMS study. All subjects were right-handed according to the Edinburgh Handedness Inventory (25) and gave informed consent before participation. Exclusion criteria for participation were (1) drug or alcohol addiction, (2) tiredness as a pharmaceutical side effect, (3) diagnosis of a comorbid neuropsychiatric disorder, and (4) any contraindication to receiving TMS as listed in the guidelines of the International Federation of Clinical Neurophysiology (26). Four participants (two patients and two healthy subjects) had to be excluded because their motor threshold was too high to be stimulated using the small coils; hence, the data of 12 patients with RR-MS and 12 healthy controls were analyzed. The group data of the patients are listed in Table 1. The study was approved by the Regional Committee on Health Research Ethics of the Capital Region in Denmark.

**Table 1:** Group data.

		**MSP *n* = 12**		**HC *n* = 12**	
	**Mean ± SD**	**Range**	**Mean ± SD**	**Range**	
Age (years)	39 ± 9	(26–52)	35 ± 10	(23–52)	
Gender (male:female)	6:6		5:7		
EDSS score (median)	2.3		NA		
Disease duration (years)	6 ± 4	1–14	NA		
FSMC total score	41 ± 16	(11–62)	27 ± 8	(20–46)	<0.01
FSMC motor score	27 ± 12	(10–45)	12	(10–23)	<0.01
FSMC cognitive score	31 ± 12	(10–46)	14	(10–24)	<0.01
PASAT score	59 ± 2.4	(51–60)	60	(60)	n.s
SDMT score	0.98 ± 0.1	(0.98–1)	(0.99 ± 0.005)	(0.98–1)	n.s
RMT (Resting Motor Threshold)	68 ± 9	(47–83)	66 ± 12	(44–90)	

*The group characteristics of both groups (MS, Multiple Sclerosis; HC, Healthy Controls), displaying the mean and standard derivation (SD) as well as the range of basic demographic and clinical measures, where appropriate. NA, not applicable*.

All MS patients were recruited from the Danish Multiple Sclerosis Centre (DMSC) at Rigshospitalet in Copenhagen. Patients with a relapsing-remitting disease course, without clinical or radiological relapses for at least 3 months and Expanded Disability Status Scale (EDSS) scores of ≤ 3.5 were selected for the study. Fatigue was assessed with the Fatigue Scale for Motor and Cognitive Functions (FSMC) (27). Cognitive functioning was assessed using the symbol digit modality test (SDMT) and the Paced Auditory Serial Addition Task (PASAT) (28, 29). Participants included in this study were part of a larger multimodal neuroimaging project conducted at the Danish Research Centre for Magnetic Resonance (DRCMR). While all MS patients who participated in the neuroimaging project were offered the opportunity to take part in the additional TMS testing day, only 14 consented to taking part in this TMS sub-study. This low number was likely due to the extensive test protocol that all participants had already undergone prior to the TMS experiment. All participants had completed three testing days, including clinical assessments, structural and functional magnetic resonance imaging, and electroencephalography. These data are reported elsewhere (16).

### Experimental Procedure

**Figure 1:**
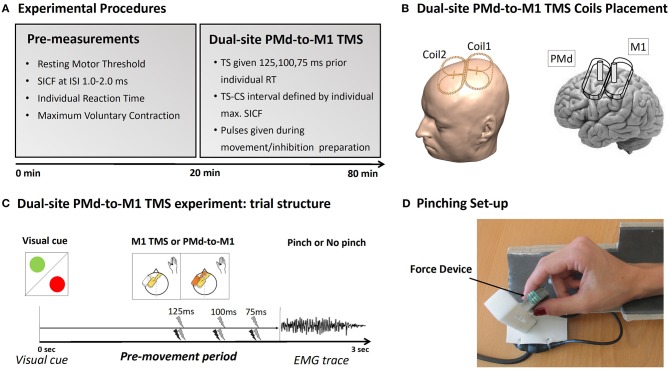
Experimental procedure. **(A)** Description of the experimental TMS procedure. **(B)** Placement of the mini-coils used during the experiment. **(C)** Plot of the Pinch-NoPinch experiment: subjects were instructed by a visual cue to either perform a pinch movement (green circle) or to refrain from preforming the movement (red circle). Single-site TMS over M1 and dual-site TMS over PMd-M1 were given 125, 100, or 75 ms before movement onset. **(D)** The pinch grip device used during the experiment.

The experimental procedures are illustrated in Figure 1. At the beginning of the experiment, short-interval intracortical facilitation (SICF) at ISIs between 1.0 and 2.0 ms was used to probe the intracortical facilitatory circuits that generate indirect waves (I-waves). This was primarily done to determine the optimal inter-stimulus interval (ISI) for the subsequent main experiment (dsTMS) (23, 24). The individual interval that elicited the strongest SICF was chosen as individual ISI and ensured that the timing between the CS and the TS was set so that that the first I-wave elicited by the TS over M1 coincided with the CS over the PMd [(see (23, 24)]. The intervals that elicit the strongest ipsilateral PMd-M1 facilitation suggest that the ipsilateral PMd-M1-HAND paradigm targets I-wave circuits: Pmd-M1facilitation peaks at around 1.2, 2.4, and 4.0 ms and thereby closely mirrors the three I-wave peaks observed during short intracortical facilitation (SICF).

We also determined the individual mean reaction time during the Pinch-No Pinch task to adjust the timing of the TS during the main experiment. In the main experiment, corticospinal excitability and PMd-to-M1 connectivity were assessed during a visually cued Pinch-NoPinch task. The task was performed with the tips of the thumb and index finger of the right hand. Single- and dual-site TMS trials were intermixed, allowing the influence of action context on both corticomotor excitability and PMd-to-M1 connectivity to be tested in the same experiment. It is important to stress that the Pinch-NoPinch task used in this experiment is different from a classical Go-NoGo task since the pinch task requires a slight isometric force even in the NoPinch condition to keep the sensor in place. Hence the NoPinch condition may not reflect a complete inhibition but instead “downscaling” of pinch force while maintaining the current pinch position.

### Experimental Setup

During the experiment, the participants sat in a comfortable chair with an arm- and headrest. While determining the SICF, the arms were placed on the armrest, entirely at rest. During the main experiment, the participants also had their arm on the armrest but held a force device between their right index finger and thumb. The Pinch-NoPinch task required the participants to react to a green or red circle. The color of the circle indicated a pinch (green) or NoPinch (red) trial. Participants had to react to the visual cue by either increasing pinch force or by keeping the force sensor in their hand without increasing pinch force. Before the task, participants were instructed to press the device quickly and with maximal force every time the green circle appeared. This was done to determine the individual reaction time and force level. Throughout the experiment, visual feedback reflecting the applied force was given.

To quantify MEPs both during SICF and the main experiment, the electromyographic (EMG) activity of the right first dorsal interosseus (FDI) muscle was recorded using surface Ag/AgCl electrodes in a bipolar montage. The signal was amplified by the factor 1,000 (D360, Digitimer, Hertfordshire, UK), band-pass filtered between 2 and 2,000 Hz and digitized at a frequency of 5,000 Hz (CED Micro 1401, Cambridge Electronic Design, Cambridge, UK). Signal 4 was used for data acquisition and further analysis (Signal Version 4 for Windows, Cambridge Electronic Design, Cambridge, UK).

### Determination of Individual I-Wave Peak and Pinch Reaction Time

#### SICF

Since I-wave latencies and I-wave facilitation display considerable inter-individual variability (23), we chose to probe PMd-to-M1 connectivity at the inter-pulse interval reflecting the individual I_1_-wave peak in each participant. The individual I_1_-wave peak was determined using a SICF protocol. The intensity of the TS was set to induce MEPs of about 1 mV in the relaxed right FDI muscle, while the intensity of the CS was set at 90% of Resting Motor Threshold (RMT). Six different inter-stimulus intervals (ISIs) ranging from 1.0 to 2.0 ms (steps of 0.2 ms) were repeated 10 times in pseudorandom order. Trials were averaged for each ISI, and the ISI with the greatest facilitation was used in the subsequent main experiment as ISI between M1-HAND and PMd stimulation. The SICF-curve was measured using an MC-B70 coil connected to a MagPro stimulator (MagVenture, Farum, Denmark). Note that we used a different coil from the ones used for the dual site-TMS session since the small Mag&More coils required for ipsilateral PMD-M1 stimulation did not allow two consecutive pulses to be fired through the same coil at the short inter-pulse intervals required by the SICF. The RMT for both coil types was determined in the relaxed FDI muscle using the Parameter Estimation in Sequential Test (PEST) method (30, 31).

#### Pinch-Task Reaction Times

To ensure that stimulation in the ds-TMS experiment was given at comparable time points during movement preparation, the individual Reaction Time (RT) for the Pinch trials of the Pinch-NoPinch task was measured before the main experiment. RT was defined as the time point at which participants started to increase the force of their contraction 10% above baseline toward the target. TMS during the main experiment was timed 125, 100, or 75 ms before the individual averaged response time.

The Maximum Voluntary Contraction (MVC) was also calculated for each participant in order to set the individual force level required during the Pinch-NoPinch task. RT and MVC were calculated from averaging 25 Pinch-NoPinch trials without TMS.

### Main Experiment

During the main experiment, two mini-coils (56 × 104 mm^2^) connected to a PowerMAG Research 100 stimulator (Mag&More GmbH, Munich, Germany) were used. The M1-HAND pulse was set to produce an MEP of 0.5 mV amplitude. This intensity was chosen because the small coils were not strong enough to reliably elicit an MEP of 1 mV. The intensity was determined using the PEST method (30, 31) while the hand was already in position for the pinch task. The M1 coil was placed tangentially to the scalp at a 45° angle over the functional hotspot for the right FDI. The intensity for the PMd coil was set to 90% of the RMT, and the location of the PMd coil was determined by physically attaching it to the M1 coil (24). The TS to M1 was given 125, 100, or 75 ms prior to individual movement onset (24). In half of all trials, a conditioning pulse to PMd followed the test pulse. The ISI between both pulses was set to the individual I_1_-wave peak determined by the SICF. Hence, the experiment tested corticospinal excitability (TS-only trials) and PMd-M1 connectivity (PMd-M1 pairs) in six different conditions (Action Context: Pinch/NoPinch; Action Timing: 125, 100, 75 ms prior to movement onset). Trials were pseudo-randomly intermixed, and, for each condition, 12 MEPs were recorded, leading to 144 trials per subject. Neuronavigation (TMS Navigator, LOCALITE, St. Augustine, Germany) of the M1 coil (and, by proxy, of the physically attached PMd coil) allowed precise monitoring of the coil position throughout the experiment.

### Statistical Analysis

All statistical analyses were conducted using IBM SPSS Statistics (version 22 for Windows) with the significance threshold set at *P* < 0.05. The Kolmogorov–Smirnov test and Mauchly test were performed to verify the assumptions of normality and sphericity in the distribution of all the data. The Greenhouse-Geisser correction method was used to correct for non-sphericity. Group matching regarding age, gender, and RMT were tested with independent sample *t*-test and χ^2^ depending on the data type. Bonferroni correction was applied to correct for multiple comparisons. Data are given as mean ± standard error of the mean (SEM).

#### Short Latency Intracortical Facilitation

All individuals showed their peak facilitation between 1.2 and 1.6 ms. While the SICF was primarily done to individualize the ISI in the main experiment, intracortical facilitation was also tested for group differences, focusing on the ISI at which the SICF peaked in each subject. MEP amplitudes (normalized to the test stimulus) at the peak ISIs were tested for group differences using an independent sample *t*-test.

#### Behavioral Data

To evaluate the difference in Reaction Time (RT) during the Pinch-NoPinch task, we used a mixed-effects model ANOVA with *Group* (two levels: MS and HC) as the between-subject factor and *TMS* (two levels: TS-only/TS-CS) and *Time* (three levels: 75/100/125 ms prior to average RT) as within-subject factors.

#### Corticospinal Excitability During Pinch-NoPinch Task

To investigate corticospinal excitability during a Pinch-NoPinch task, all TS-only trials were analyzed in a mixed-effects ANOVA using the normalized MEPs as the dependent variable, with *Group* (two levels: RR-MS and HC) as the between-subject factor and *Task* (two levels: Pinch/NoPinch) and *Time* (three levels: 75/100/125 ms prior to average RT) as within-subject factors. *Post-hoc* tests following significant main effects or interaction effects were Bonferroni-corrected.

#### PMd-M1 Connectivity During Pinch-NoPinch Task

To investigate ipsilateral PMd-M1 connectivity, all TS-CS trials were analyzed using a mixed-effects ANOVA with *Group* (two levels: MSP/HC) as the between-subject factor and *Task* (two levels: Pinch-NoPinch) and *Time* (three levels: 75/100/125 ms prior to average RT) as within-subject factors. MEPs were normalized to the single pulses given at the same Time and Task.

#### Correlations

To test whether abnormal PMd-M1 connectivity in patients correlates with fatigue severity (motor score), we performed Pearson correlation analysis. Since we expected task-dependent modulation of PMd-M1 connectivity to increase closer to movement initiation or inhibition, we calculated the change in normalized PMd-conditioned MEP-size when approaching Pinch and NoPinch execution (Δ 100–75 ms) and correlated these values with individual FCMS severity scores. To test for the timing specificity of our results, we also calculated correlations between PMd-conditioned MEPs at a different delta interval (Δ 125–100 ms). Bonferroni correction was used to obtain family-wise error-corrected p-values where appropriate.

## Results

### Basic Group Characteristics

Age and sex were not significantly different between healthy controls and patients (age: t(22) = −0.854, *p* = 0.402; sex: χ_(1)_ = 0.168, *p* = 0.682). Neither were cognitive test scores (PASAT & SDMT), basic neurophysiological parameters like RMT, or the stimulator output used to achieve 1 mV during SICF or 0.5 mv during the main experiment (all *p* > 0.40).

### Short Latency Intracortical Facilitation

**Figure 2:**
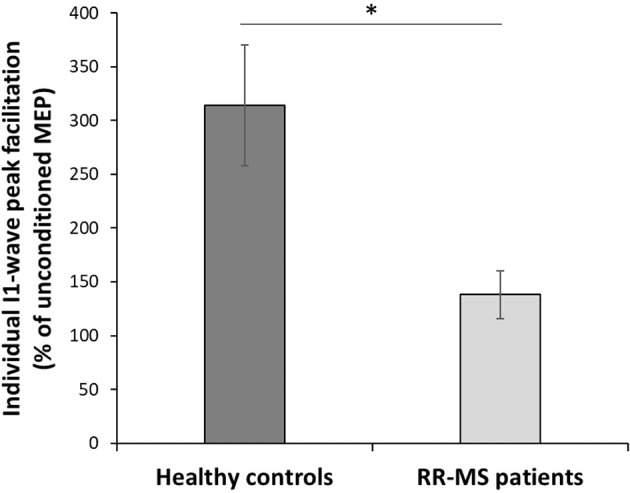
Short Intracortical Facilitation (SICF). Individual most effective ISIs normalized with respect to the TS. Multiple sclerosis patients show a significantly lower amplitude of the I1-wave peak facilitation with respect to the HC (t(22) = 2.396; *p* = 0.026); ^*^p < 0.05.

Test stimulus size during the SICF was not significantly different between groups (t(21) = −1.616; *p*= 0.12). All participants did show SICF in at least one interval, and all participants had their most responsive interval (e.g., the interval with the highest facilitation) between 1.2 and 1.6 ms (Table 2). To test for differences in intracortical facilitation between groups, the MEP amplitude for the most effective SICF interval was normalized to the test pulse in each participant. A *t*-test comparing the optimal SICF between groups showed that RR-MS patients showed significantly less facilitation than healthy volunteers (t(22) =2.39; *p* = 0.026) (Figure 2).

**Table 2:** TMS group data.

	**MSP** ***n*** **=** **12**			**HC** ***n*** **=** **12**		
	**Mean**	**SD**	**Range**	**Mean**	**SD**	**Range**
SICF						
ISI	1.2			1.2		
RMT	34.67	4.60	28–43	34.42	6.27	(24–47)
dsTMS						
RT (ms)	359.60	41.50	(274.4–433.2)	397.94	77.48	(301.2–526.3)

*Basic neurophysiologic measures of both groups (MS = Multiple Sclerosis; HC = Healthy Controls), displaying the mean and standard derivation (SD) as well as the range of basic demographic and clinical measures, where appropriate. ISI (interstimulus interval) denotes the most effective inter-stimulus interval during the SICF (Short Intracortical Facilitation) protocol and RMT the average Resting Motor Threshold. RT denotes the reaction time to initiate a pinch force during the Go condition of the PinchNoPinch task*.

### Behavioral Data

We tested whether either group membership (RR-MS vs. HC), TMS condition (TS vs. TS-CS), or TMS timing (75/100/125 ms) influenced the performance during the Pinch-NoPinch task. A mixed-effects ANOVA with RT as the dependent variable showed that none of the factors had a significant influence on RT (Time: F_(1.073, 23.597)_ = 1.458, *p* = 0.242; Group (F_(1, 22)_ = 1.454, *p* = 0.241; Condition: F_(1, 22)_ = 0.499, *p* = 0.487). Also, none of the interactions were significant (*p* > 0.2).

### Single-Pulse TMS Data

**Figure 3:**
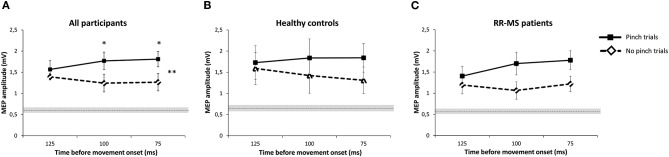
Pinch-NoPinch task. **(A)** Pre-movement facilitation of the MEP amplitude during the Pinch/NoPinch task, averaged over all participants. The dotted, horizontal line indicates the mean value of the baseline MEP (0.63 mV) with standard error as a gray shadow (0.06). Error bars indicate standard error. **(B)** Mean MEP amplitudes of the HC group during the task. The dotted horizontal line indicates the mean value of the baseline MEP for HC (0.65 mV± 0.09). Error bars indicate standard error. **(C)** Mean MEP amplitudes of the MS group during the task. The dotted horizontal line indicates the mean value of the baseline MEP for MS patients (0.61 mV ± 0.09). Error bars indicate standard error. ^*^p < 0.05.

To test for action context-dependent modulations of corticospinal excitability, a mixed-effects ANOVA was calculated, with the MEPs elicited by the TS during the Pinch-NoPinch task as the dependent variable. The ANOVA indicated that the action context modulated corticospinal excitability in both groups, with higher MEPs during the Pinch-trials (main effect of Task: F_(1, 22)_ =7.068, *p* = 0.014). Mean MEP amplitude evoked by TS in the Pinch and NoPinch conditions was consistently larger than the 0.5 mV MEP amplitude evoked by the TS at rest, as determined before the start of the experiment. A significant Time × Task interaction further indicated that corticospinal facilitation increased when the TS was given closer to Pinch onset and dropped (though not under baseline) when the TS was given closer to the (imaginary) in the No-Pinch condition (Figure 3A). (F_(2, 44)_ = 4.123, *p* = 0.023). The lack of a significant *Group* effect indicated that there was no significant difference in time-dependent modulation of corticospinal excitability between the groups (Figures 3B,C). This indicated that MS patients modulated corticospinal excitability in a task-dependent fashion and, to a degree, were comparable to healthy controls (F_(1, 22)_ = 0.320, *p* = 0.578) (Figure 3C).

### Dual-Site TMS Data: Modulations of Premotor-M1 Connectivity

**Figure 4:**
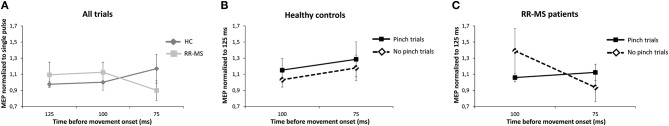
Dual-site TMS (dsTMS) protocol during the Pinch-NoPinch Task. Pre-movement MEP amplitude modulation during the Pinch-NoPinch task. Each line represents the mean amplitude of the MEPs elicited by dsTMS, normalized to the mean MEP amplitude elicited by the single pulse at the same time interval. Error bars indicate standard error. **(A)** Modulation of MEP amplitude between HC and MS patients over time. **(B)** Change in PMd-M1 modulation when getting closer to (potential) movement onset for the HC-group, normalized to the mean MEP at 125 ms. **(C)** Change in PMd-M1 modulation when getting closer to (potential) movement onset for the MS-group normalized to the mean MEP at 125 ms.

A mixed-effects ANOVA calculated on the normalized MEPs elicited by dual-site TMS did not reveal a significant effect of *Group, Task*, or *Time* on PMd-M1 connectivity. However, there was a trend toward a *Time* × *Group* interaction (F_(2, 44)_ = 2.760, *p* = 0.074; Figure 4A). *Post-hoc* tests did not find significant group differences but indicated that the difference between groups was largest closest to movement onset (75 ms, *p* = 0.1) (Figures 4A–C).

### Dynamic Modulation of PMd-M1 Connectivity as a Marker of Motor Fatigue

**Figure 5:**
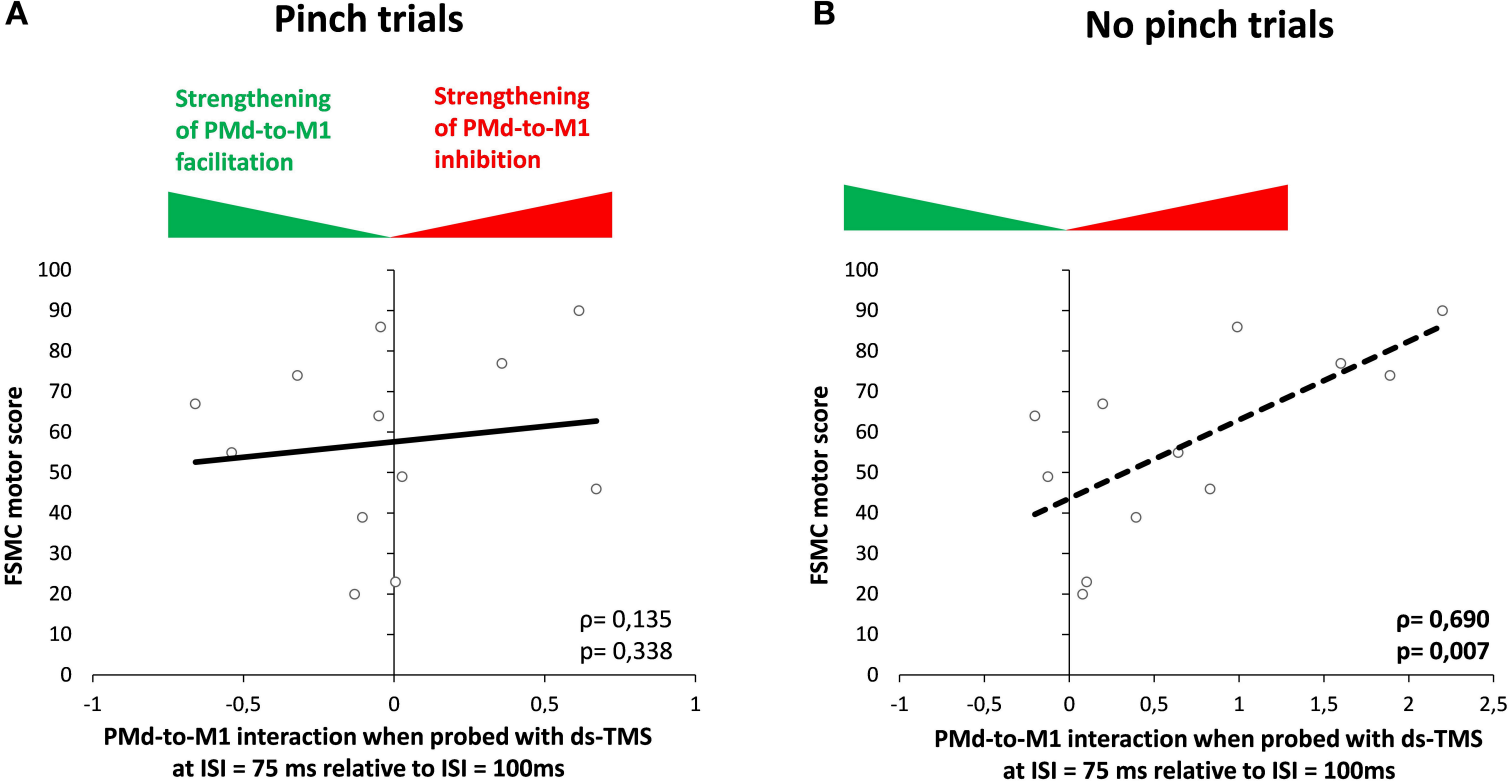
Correlation analysis. Correlation between FSMC motor score and the normalized modulation of MEP from 100 to 75 ms (Δ score) during the Pinch **(A)** and NoPinch **(B)** tasks in the MS group. M1, primary motor cortex; PMd, dorsal premotor cortex.

In patients, we found a significant positive correlation between increasing PMd-M1 inhibition, indicated by ΔMEP (100–75 ms), and the FSMC motor score for the NoPinch trials but not for the Pinch task (NoPinch: *r* = 0.69, *p* = 0.007; Bonferroni-corrected *p* = 0.028; Pinch: *r* = 0.21, *p* = 0.50; Figure 5). While a similar trend could be observed for ΔMEP (125–100 ms), NoPinch correlations for this interval were weaker and did not survive Bonferroni correction (NoPinch: *r* = 0.587; *p* = 0.022; Bonferroni-corrected *p* = 0.08; Pinch *r* = −0.175; *p* = 0.293). In healthy subjects, no significant correlations could be observed (*p* > 0.2). There was also no correlation between the dynamic modulation of corticospinal excitability as revealed by single-pulse TMS and individual FSMC scores.

## Discussion

Using single-pulse and paired-pulse TMS, we studied the dynamic modulation of motor cortical excitability during a cued Pinch-NoPinch task in patients with RR-MS. Our measurements yielded two main findings: First, patients showed a normal modulation of corticospinal excitability as well as ipsilateral premotor-to-motor cortical drive during the RT period of Pinch and NoPinch trials. Second, patients showed a tendency toward attenuation of the premotor-to-motor drive toward the end of the RT period in NoPinch trials. This attenuation in functional premotor-to-motor connectivity scaled positively with the amount of motor fatigue in patients. Mean response times did not differ between groups or TMS conditions, indicating that our TMS measurements during the RT period were not confounded by relative differences in the timing of TMS with respect to the appearance of the cue. The comparable RTs also exclude the possibility that TMS prolonged the RT period in patients with MS relative to healthy controls (32). Importantly, measures of cognitive functioning did not differ between groups indicating that executive control processes like information processing efficiency and speed were not affected in the tested patients (33).

### Modulation of Corticospinal Excitability During the RT Period

Single-pulse TMS applied to the left M1-HAND revealed a comparable modulation of corticospinal excitability in patients with RR-MS and healthy controls. Both groups showed a substantial increase in corticospinal excitability across all ISIs and experimental conditions. This indicates that the RT period was characterized by “global” corticospinal facilitation. It also implies that the decision to refrain from further action in NoPinch trials did not require global inhibition but rather a gradual downscaling of facilitation.

In addition to global MEP facilitation, there was a time-dependent modulation of corticospinal excitability during the RT period, caused by a divergence of corticospinal excitability in Pinch and NoPinch trials toward the end of the RT period. Corticospinal excitability increased further if the visual cue instructed participants to pinch. Conversely, corticospinal excitability showed a relative decrease during the RT period if the visual cue required participants to refrain from pinching. This finding agrees with previous studies demonstrating context-dependent regulation of excitability using a classical Go-NoGo task (34, 35), even though the classical NoGo condition leads to active suppression of corticospinal excitability rather than the relative downscaling of facilitation observed during NoPinch trials in our study.

The differential effect of action context on the dynamic regulation of corticospinal excitability was similar in patients with MS and healthy controls. This observation is in contrast with previous single-pulse TMS studies, which found attenuated pre-movement facilitation of MEP amplitude in simple cued RT tasks (14, 15). The discrepant findings highlight that the specific movement context may be pivotal in detecting disease-dependent changes in pre-movement excitability. Indeed, differences in the motor context can reconcile the apparently diverging findings. The simple, cued RT-task used in previous studies required a rapid initiation and release of the same action across all trials based on a very simple cue-response mapping rule. In contrast, cue-response mapping was more complex in the Pinch-NoPinch RT task used in the present study and required fine control of grip force levels during pinching. We argue, therefore, that a disease-dependent reduction of pre-movement facilitation does not generalize across motor tasks. Rapid boosting of corticospinal excitability during simple externally cued motor actions appears to be impaired in MS and associated with motor fatigue (14, 15). In contrast, more finely tuned and bi-directional regulation of corticospinal excitability during deliberate choices to act (to pinch) or not to act (not to pinch) may be unaffected—at least in moderately affected patients with RR-MS. Another factor that may have helped patients to reach standard pre-movement modulation was the overall corticomotor facilitation induced by the task: the substantial increase in corticospinal excitability throughout all ISIs and experimental conditions indicates a “global” corticospinal facilitation and suggests that the decision to refrain from further action in NoPinch trials did not require global inhibition but rather a downscaling of facilitation.

### Ipsilateral Premotor-to-Motor Drive

Our dsTMS measurements revealed no differences in the ipsilateral premotor-to-motor drive between patients with RR-MS and healthy controls. In both groups, the CS given to the left PMd elicited no extra facilitation of the MEPs evoked with a TS over ipsilateral M1-Hand. This was the case when participants were about to initialize a pinch or refrained from executing a pinch. The results suggest that ipsilateral premotor-motor drive may have already been saturated during the RT period, which may have prevented the premotor CS from further increasing premotor-to-motor facilitation.

The dsTMS results show that the relative strength of effective ipsilateral PMd-to-M1-HAND facilitation was constant throughout the task and was not consistently altered by MS. However, subtle alterations in premotor-to-motor connectivity were observed in patients with RR-MS at the last ISI, which was closest to the end of the RT period. While healthy controls displayed a non-significant faciliatory premotor-motor effect in both trial conditions, MS patients showed a slightly inhibitory influence of the ipsilateral premotor CS on corticospinal excitability during NoPinch trials. Correlation analysis demonstrated that the individual magnitude of premotor-to-motor inhibition scaled linearly with the severity of motor fatigue in MS patients. The more severe the motor fatigue, the stronger the inhibitory drive from ipsilateral PMd to M1-HAND was approaching a NoPinch event. This relationship was not present in the Pinch trials and was not found in the healthy control group. This finding indicates that patients who are more affected by motor fatigue show stronger downregulation of effective PMd-to-M1 connectivity in a context of movement inhibition and this may indicate a less effective mode of fine tuning the PMd-to-M1 drive.

### Short-Latency Intracortical Facilitation

Using paired-pulse TMS applied to the precentral gyrus, we replicated a previous paired-pulse TMS study showing a relative reduction in SICF in MS patients relative to healthy controls (9). However, unlike the previous study, we were not able to observe a correlation between individual reduction in SICF and disease severity (9).

### Caveats

The presented study was subsidiary to a larger project (16), and hence the number of participants was determined by the retention rate at which participants agreed to participate in the additional testing day. While previous studies on the action context-dependent modulation of premotor-motor connectivity (24) have used comparable sample sizes, it is possible that the sample of 12 participants per group reduced the chance of detecting subtle between-group differences. Future studies are needed to determine whether the reported trend for a general *Time* × *Group* interaction in premotor-M1 connectivity would become significant in a better-powered study.

## Conclusions

Our data suggest that pre-movement facilitation in M1 is not impaired in MS patients if probed in a complex context of action preparation and action inhibition. However, we were able to demonstrate subtle abnormalities in premotor-motor connectivity, where decreasing PMd-M1HAND facilitation during movement inhibition predicted the severity of fatigue scores in MS patients. This may indicate that patients suffering from motor fatigue require stronger modulation of their PMd-M1 drive to implement movement inhibition. Our findings challenge disease-dependent modulation of corticospinal excitability and indicate that functional premotor-motor connectivity may be important in understanding the pathology of fatigue in MS.

## Data Availability Statement

The datasets generated for this study are available on request to the corresponding author.

## Ethics Statement

The studies involving human participants were reviewed and approved by Regional Committee on Health Research Ethics of the Capital Region in Denmark. The patients/participants provided their written informed consent to participate in this study.

## Author Contributions

ERu, RD, ERa, AK, and HS designed the study. ERu, OS, and AK collected the data. ERu, RD, KM, and KA analyzed the data. ERu, ERa, RD, AK, KM, and HS wrote the paper.

## Conflict of Interest

HS has received honoraria as speaker from Sanofi Genzyme, Denmark, and Novartis, Denmark, as a consultant from Sanofi Genzyme, Denmark, and as senior editor (NeuroImage) from Elsevier Publishers, Amsterdam, The Netherlands. He has received royalties as a book editor from Springer Publishers, Stuttgart, Germany. AK is a shareholder and co-founder of BrainChild Technologies, LLC. OS has received travel funding from Biogen Idec. All conflicts of interest are unrelated to this work. The remaining authors declare that the research was conducted in the absence of any commercial or financial relationships that could be construed as a potential conflict of interest.

## Abbreviations

CNS: Central Nervous System
CS: Conditioning Stimulus
DMSC: Danish Multiple Sclerosis Centre
dsTMS: dual-site TMS
EDSS: Expanded Disability Status Scale
EMG: Electromyography
FDI: First Dorsal Interosseous
FSMC: Fatigue Scale for Motor and Cognitive Functions
HC: Healthy Controls
ISI: Interstimulus Interval
M1: Primary Motor Cortex
M1_HAND_: Primary Motor Hand Area
MCV: Maximum Voluntary Contraction
MEP: Motor Evoked Potential
MS: Multiple Sclerosis
PASAT: Paced Auditory Serial Addition Test
PEST: Parameter Estimation in Sequential Test
PMd: dorsal Premotor Cortex
PMd-M1: Premotor-Motor
RMT: Resting Motor Threshold
RR: Relapsing-Remitting
RT: Response Time
SD: Standard Error
SDMT: Symbol Digit Modalities Test
SEM: Standard Error of the Mean
SICF: Short Intra Cortical Facilitation
sMRI/fMRI: structural and functional Magnetic Resonance Imaging
TMS: Transcranial Magnetic Stimulation
TS: Test Stimulus.
